# Jose Maria Peralta, M.D., D.V.S.

**Published:** 1897-01

**Authors:** 


### NECROLOGY.

JOSE MARIA PERALTA, M.D., D.V.S. The sad news reaches us by cable of the death of Dr. Peralta, in Cuba, where he had gone to aid the Cubans in their struggle for freedom. While engaged in some strategic work in behalf of those with whom his sympathies and aid were cast he was shot down by a Spanish soldier. Dr. Peralta was a graduate of the American Veterinary College of New York City, Class of 1892, and of Jefferson Medical College, Philadelphia. He served as a resident physician of St. Joseph's Hospital, Lancaster, Pa., for a period of time after the completion of his studies, and represented his country, Costa Rica, at the World's Fair in 1893. In these positions he won many warm friends, who, with those who had the pleasure of his acquaintance and the warm friendship of his sunny disposition, will with one accord be distressed at his untimely and sad death.

